# [Corrigendum] RIPK4/PEBP1 axis promotes pancreatic cancer cell migration and invasion by activating RAF1/MEK/ERK signaling

**DOI:** 10.3892/ijo.2025.5822

**Published:** 2025-11-24

**Authors:** Zi-Hao Qi, Hua-Xiang Xu, Shi-Rong Zhang, Jin-Zhi Xu, Shuo Li, He-Li Gao, Wei Jin, Wen-Quan Wang, Chun-Tao Wu, Quan-Xing Ni, Xian-Jun Yu, Liang Liu

Int J Oncol 52: 1105-1116, 2018; DOI: 10.3892/ijo.2018.4269

Following the publication of the above paper, it was drawn to the Editor's attention by an interested reader that the middle and right-hand protein blots shown for the RIPK4 data in Fig. 2B (relating to the PANC-1-Rsh1 and PANC-1-Rsh2 experiments) were strikingly similar to western blot data shown in Fig. 3B for the RAF-1 data (and the same PANC-1-Rsh1 and PANC-1-Rsh2 experiments), albeit the bands were presented with different exposures/a change in contrast, also with apparent horizontal flipping and vertical resizing. Upon contacting the authors, they realized that errors had been made during the assembly of the experimental images presented in Fig. 3B. These errors were likely to have resulted from oversights made during the process of data consolidation and figure assembly; specifically, this led to the inadvertent use of incorrect images for the RAF-1 western blot results in both the PANC-1 cell line (as was correctly identified by the interested reader on PubPeer) and in the Capan-1 cell line (which the authors identified themselves upon performing their own subsequent review). The authors were also able to present photos of the raw, unedited versions of the gels to the Editorial Office.

A revised version of Fig. 3, now showing the correct data for the RAF-1 blots for both the PANC-1 and Capan-1 cell lines, as specified above, is shown on the next page. The authors confirm that the errors made in assembling Fig. 3 did not have a major impact on the conclusions reported in the above article, and they thank the Editor of *International Journal of Oncology* for allowing them the opportunity to publish a Corrigendum. Furthermore, all the authors agree to the publication of this Corrigendum, and apologize to the readers for any inconvenience caused.

## Figures and Tables

**Figure 3:**
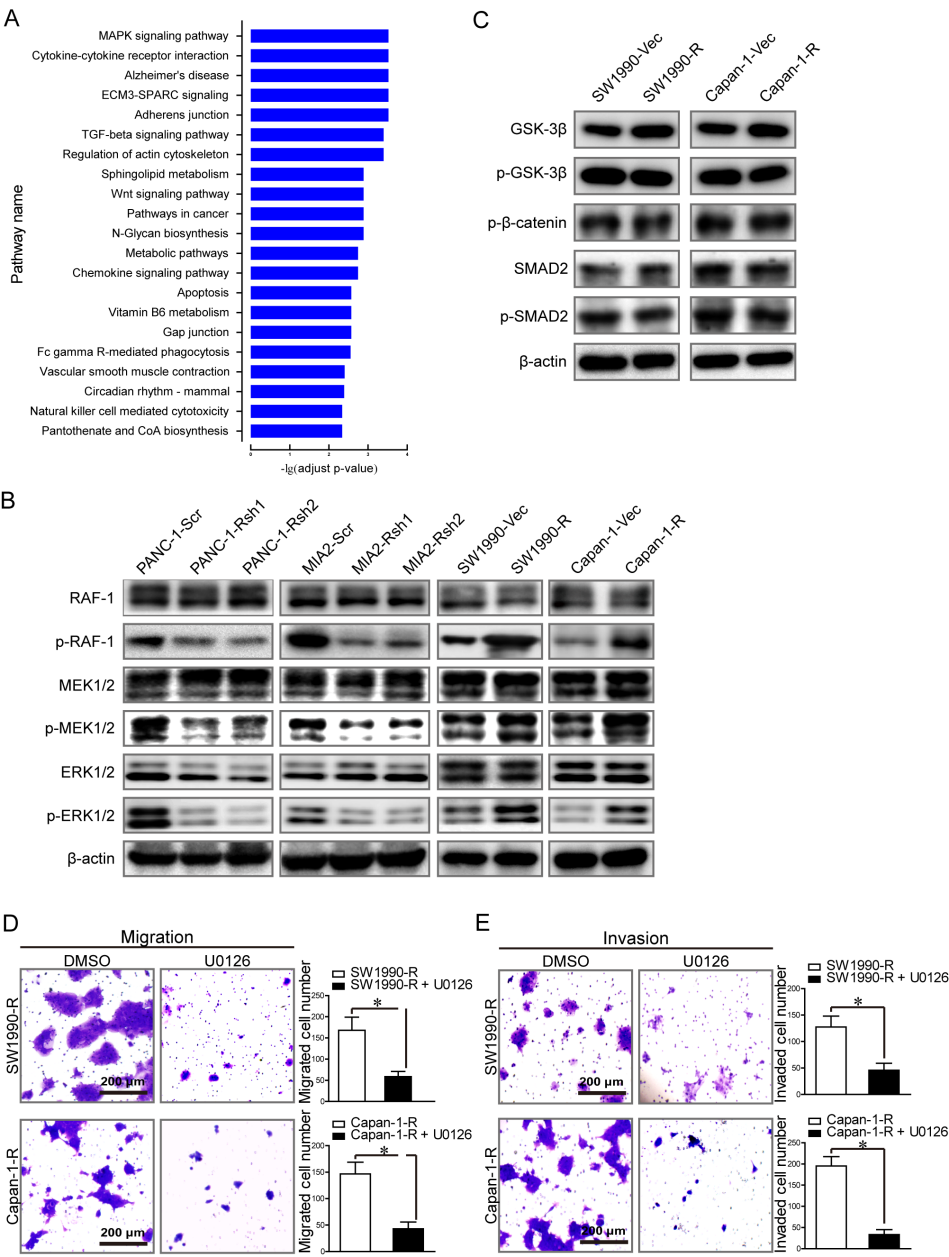
Receptor-interacting protein kinases 4 (RIPK4) promotes pancreatic cancer tumor cell metastasis via the RAF1/MEK/ERK pathway. (A) Pathway enrichment analysis of differentially expressed genes using the KEGG pathway database. (B) The levels of total and phosphorylated RAF1, MEK1/2 and ERK1/2 in pancreatic cancer cell lines in which RIPK4 was overexpressed or knocked down. (C) The levels of SMAD2, p-SMAD2, GSK-3β, p-GSK-3β and p-β-catenin in pancreatic cancer cell lines in which RIPK4 was overexpressed were detected by western blot analysis. (D and E) The effects of blocking RAF1/MEK/ERK signaling on pancreatic cancer cell (D) migration and (E) invasion were determined by Transwell assays using RIPK4-overexpressing Capan-1 and SW1990 cell lines. The numbers of migrating or invading cells were calculated, and the quantification of 3 randomly selected fields is shown in the histogram. *P<0.05.

